# Influence of primary care practices on patients’ uptake of diabetic retinopathy screening: a qualitative case study

**DOI:** 10.3399/bjgp14X680965

**Published:** 2014-07-25

**Authors:** Antje Lindenmeyer, Jackie A Sturt, Alison Hipwell, Irene M Stratton, Nidal al-Athamneh, Roger Gadsby, Joseph Paul O’Hare, Peter H Scanlon

**Affiliations:** School of Health and Population Sciences, University of Birmingham, Birmingham.; Florence Nightingale School of Nursing and Midwifery, King’s College London.; Division of Health Sciences, Warwick Medical School, Warwick.; Gloucestershire Royal Hospital, Gloucestershire Hospitals NHS Foundation Trust, Gloucester.; Division of Health Sciences, Warwick Medical School, Warwick.; Division of Health Sciences, Warwick Medical School, Warwick.; Division of Health Sciences, Warwick Medical School, Warwick.; Gloucester Diabetic Retinopathy Research Group, Cheltenham General Hospital, Gloucestershire Hospitals NHS Foundation Trust, Cheltenham.

**Keywords:** diabetic retinopathy, interprofessional relations, mass screening, primary care, qualitative research

## Abstract

**Background:**

The NHS Diabetic Eye Screening Programme aims to reduce the risk of sight loss among people with diabetes in England by enabling prompt diagnosis of sight-threatening retinopathy. However, the rate of screening uptake between practices can vary from 55% to 95%. Existing research focuses on the impact of patient demographics but little is known about GP practice-related factors that can make a difference.

**Aim:**

To identify factors contributing to high or low patient uptake of retinopathy screening.

**Design and setting:**

Qualitative case-based study; nine purposively selected GP practices (deprived/affluent; high/low screening uptake) in three retinopathy screening programme areas.

**Methods:**

Semi-structured interviews were conducted with patients, primary care professionals, and screeners. A comparative case-based analysis was carried out to identify factors related to high or low screening uptake.

**Results:**

Eight possible factors that influenced uptake were identified. Five modifiable factors related to service and staff interactions: communication with screening services; contacting patients; integration of screening with other care; focus on the newly diagnosed; and perception of non-attenders. Three factors were non-modifiable challenges related to practice location: level of deprivation; diversity of ethnicities and languages; and transport and access. All practices adopted strategies to improve uptake, but the presence of two or more major barriers made it very hard for practices to achieve higher uptake levels.

**Conclusions:**

A range of service-level opportunities to improve screening attendance were identified that are available to practices and screening teams. More research is needed into the complex interfaces of care that make up retinopathy screening.

## INTRODUCTION

**Figure 1.**
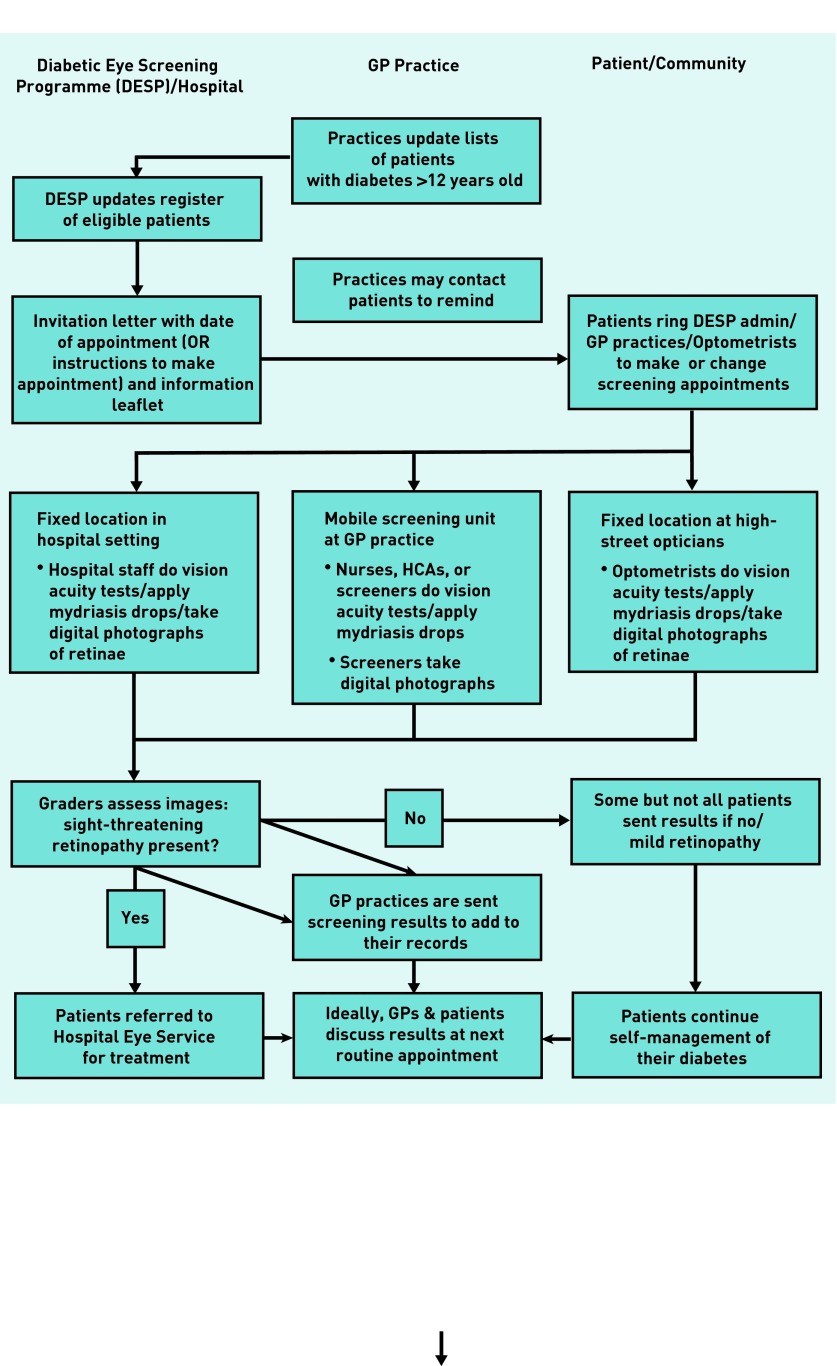
***Process of diabetic retinopathy screening.***

Diabetes is a common condition affecting one in 20 adults in the UK.^1^ Diabetic retinopathy is a microvascular complication of diabetes. Symptomless to the patient until it is in the advanced stages, it is the most common reason why people of working age in the UK become registered blind or partially sighted.^2^ The NHS Diabetic Eye Screening Programme (DESP) in England aims to reduce the risk of sight loss by offering screening for retinopathy to 2.4 million people; 84 local programmes screened 1.9 million people between February 2012 and February 2013.^3^ Figure 1 details the screening process. In most regions, the screening process is shared between the DESP and GP practices (although in some regions, screening is carried out at hospitals or high-street optometrists). While the DESP sends out invitations, arranges appointments, and provides screening staff and equipment, GP practices provide the venue; often, nurses are involved in doing vision acuity tests and administering mydriasis drops. Although the managerial aspect of screening is beyond the control of the practices, they are still judged and remunerated through the Quality and Outcomes Framework (QOF) according to their uptake rates. This necessitates cooperation between practices and the DESP, as well as involvement of practices in motivating patients to attend. This study reports the first qualitative research that aims to understand non-attendance in the context of individual GP practices. The practice provides the common link, however, multiple agencies are involved in the process and therefore multiple opportunities for uptake barriers and facilitators can be established. Improving screening uptake improves cost-effectiveness because there is a link between the number of missed appointments and levels of diabetic retinopathy.^4^

Non-attendance at screening is a risk factor for sight-threatening retinopathy.^4^,^5^ Many patients do not take up the offer of screening. In Scotland, duration of diabetes, poor control, and smoking were associated with lower uptake;^6^ in Ireland and the Netherlands, individual recommendation by a healthcare professional increased participation.^7^,^8^ Patients’ lack of awareness, psychological factors (fear and guilt), or practical obstacles, such as obtaining time off work, were also important.^9^ Patients living in the most deprived areas were less likely to attend for screening while having worse retinopathy.^10^,^11^ Uptake rates in the same area can vary between practices from 55% to 95%; practices can have a higher, or lower, uptake rate than suggested by their patient population.^12^ This provides the rationale to treat each GP practice as a case within which factors that affect screening uptake can be identified. The overall study aimed to elicit accounts from patients as well as healthcare professionals, however, this paper focuses on factors related to the practice, which include interaction with screening services and patients.

**How this fits in**

Non-attendance at diabetic retinopathy screening is associated with sight-threatening retinopathy. There is great disparity between GP practices in screening uptake rates, with patients living in the most deprived areas least likely to attend for screening. This study shows that although there were non-modifiable challenges related to patient demographics and practice location, practices were able to improve uptake by integrating screening with routine care and contacting patients. The main area for improvement identified was communication and collaboration between practice staff and regional screening teams.

## METHOD

Nine GP practices were purposively sampled in three regional screening programme areas (Table 1). In two areas screening took place at GP practices with additional fixed units at a central location/hospital, whereas in another patients were invited to make an appointment with a participating high-street optometrist. Practices were sampled to achieve a variety of backgrounds according to location (city, small town and rural), levels of deprivation (identified from Indices of Multiple Deprivation data), and screening uptake rates (from DESP data).

**Table 1.** Characteristics of participants

	**Programme area 1**	**Programme area 2**	**Programme area 3**	**Total**
**Number of practices**	4	3	2	9
**Patients (of whom low attenders)**	14 (5)	8 (1)	16 (10)	38 (16)
**Medical practice staff (GPs, nurses)**	2	3	3	8
**Administrative practice staff**	4	2	1	7
**Screeners**	4	4	1	9
**Total participants**	24	17	21	62

In each practice a range of professionals and people with diabetes were interviewed to ensure a broad spectrum of views and experiences (Table 1). Participating practices were asked to identify two members of staff with different roles who were engaged with the screening programme. Interviews were conducted in practices, at patients’ homes, or by telephone. A semi-structured topic guide aimed to capture the experience of retinopathy screening from the perspective of professionals and of patients (Boxes 1 and 2). To analyse the data, a comparative case study design was used^13^,^14^ to identify factors leading to high or low screening uptake, with each GP practice representing a case. Some of the authors conducted a thematic analysis of transcripts from one programme area each to extract factors influencing screening uptake. The research team then finalised a list of factors, which was refined and applied to the entire dataset. Factors were categorised as major enablers, minor enablers, neutral (no difference or not applicable), minor barriers, or major barriers. These factors, singly and in combination, were then systematically compared with levels of screening uptake in the practice to identify whether any factors were consistently related to uptake above or below 80% (the quality standard set by the DESP).^15^

Box 1. **Semi-structured interview schedule for professionals**

- What is your role in the diabetic retinopathy screening programme? What routines and procedures does it involve you doing?
- Do you know how many patients attend for retinal screening here? What do you think influences this?
- Do you know what information patients receive about retinal screening, what’s involved, why it’s important for them? (Patient information/preparation for retinal screening)
- From your perspective, what happens when the patient attends for screening?
- What (if anything) do you have to do if they don’t attend?
- Are you involved in informing patients about the results and any further actions?
- Are there any changes that you can suggest to improve the way your patients are invited to/informed about retinal screening and the service delivered, which would improve uptake?
- Are there any changes that you can suggest regarding (this) practice’s response to patients, following communication of screening results?
- How important do you feel retinal screening is for patients alongside their other diabetes screening activity? (Prioritisation)
- Why do you think some patients don’t attend?
- Is there anything you’d like to add that we haven’t covered in the interview?

Box 2. **Semi-structured interview schedule for patients**

- Tell us about yourself and your life at present. (Prompts: living alone/with others; working, caring, or retired; social activities)
- Can you describe a typical day living with diabetes? (Prompts: examples of how it affects your daily life? Compared to how you were before becoming ill/other people who are well?)
- Can you describe a good/bad day living with diabetes?
- Is there anything that you can do to improve your experience of living with diabetes?
- When did you last see your nurse/GP about your diabetes – and what did you talk about?
- What do you know about eye screening and diabetes?
- How did you first find out about diabetic eye screening?
- Do you know why are you asked to go?
- How do you know when and where you should go?
- Do you know what it involves? (For those who did attend screening: describe in as much detail as possible the last screening they went to)
- How does this screening fit in with the rest of your diabetes care and treatment?
- What happens after your screening – how do you find out your results?
- Have you ever needed any further treatments on your eyes? How did you find out what you needed, what your options were?
- What do you think is responsible for any deteriorating eyesight you might have? Why?
- Are there any changes to the service that you could suggest – from invitation to screening, receiving results/treatment options, etc, that would make the screening process better for you? (For example, link with opticians at annual eye test)
- What would you like to be able to do differently that would make the screening process better for you?
- What (if anything) puts you off going?
- Have you ever been invited for any other type of health screening, for example, cervical/ breast/ bowel – if so, how does it compare?
- Is there anything you’d like to add that we haven’t covered in the interview?

## RESULTS

### Participant characteristics

Through the process described above, eight main factors were identified. Five factors were modifiable, related to service and staff interactions:

1. practice communication with screening services;
2. contacting patients pre- and post-screening;
3. integration of retinopathy screening with other diabetes care;
4. focusing on the newly diagnosed; and
5. a perception among practice staff that there was a hard core of patients who would not attend screening unless they experienced symptoms.

Three factors consisted of non-modifiable challenges related to practice location. They were recognised by existing literature but confirmed in this study:

6. deprivation;
7. issues around language and culture in minority ethnic populations; and
8. accessibility of screening location.

**Figure 2.**
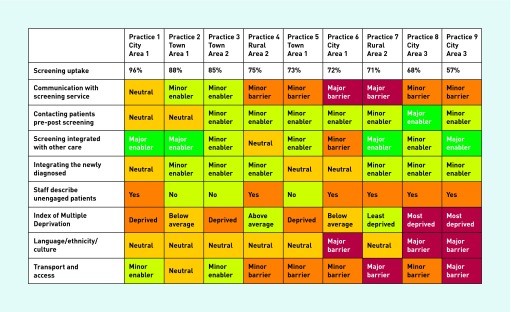
***Factors related to screening uptake.***

A summary of these factors related to practices is reproduced in Figure 2.

### Modifiable factors

Modifiable factors were linked to communication between practice staff, screeners, and patients. The greatest barriers were inflexible or incompatible administrative systems, screeners being isolated from the everyday work of the GP practice that was the focus of the uptake standard, and perceptions of defeat in relation to patients who missed many practice appointments.

#### 1. Communication with screening services

In all but the three highest performing practices, practice staff and screeners identified communication issues between practices and screening services. Centrally allocated appointments counteracted their attempts to bring patients in, especially if IT systems or administrators were perceived as inflexible. One screener outlined the pros and cons of the current system:

‘Some of the practices ... just hadn’t got any sort of system at all. So a centralised system is a good thing. But then on the other hand you will come to some practices that are really organised and they know their patients ... “oh, hang on, they’re married, so if you put them together they’ll both come in together”. And they can ... we just can’t ... with 30 000 patients you can’t organise things like that.’

(Screener, Programme area 1)

Good communication on the day led to practice staff sharing their knowledge of their patients. Several screeners said that one motivated member of staff could make a real difference, for example, by contacting patients to fill vacant appointments. Another difficulty was that practices needed to allocate a room for screeners and their mobile equipment. This arrangement was seen as superior to a van in the practice car park, but led to other practice staff feeling crowded out and screeners working in isolation if practice staff were not involved, for example, by preparing patients for screening:

*‘Someone had let me in* [in] *the morning ... I was just finished with my second patient, dilating them, and I decided to check my mobile phone and I had a missed call from the office. So I rang my admin manager and she said “I hope you’re okay, I’ve just had a phone call from the practice and they say you haven’t arrived to do your clinic”.’*

(Screener, Programme area 2)

#### 2. Contacting and motivating patients

Practice staff often described phoning patients, either in advance to remind them of their screening appointment or after they did not attend; they would then attempt to slot them in later that same week or at a central catch-up clinic. Practices with a high uptake did not see the need as their patients were ‘good at coming in’ without prompting. In practices with a large number of patients from South Asian backgrounds where members of staff spoke the same language, a GP or practice manager led a team effort to contact patients. However, this was seen as least helpful where appointments were out of the practice’s control:

*‘What I try to do is get the* [receptionists] *to ring the patients the day before to speak to them in Punjabi. The problem you’ve got then is if the patient says “well I can come at 11 instead of 9”, they can’t say yes.*

(Practice manager, Practice 6)

One practice in the programme area that used optometrists contacted patients who did not make an appointment, motivated by QOF targets:

*‘So in the first 6–8 months of the year we sort of let them get on with it and when we see them we encourage them gently. When it comes to January, February, March time we can see our data and we see that we need to work harder on this, so we’re actively ringing them.* [Nurse] *does a lot of ringing and she will ring and say “can I speak to this person? Have you been for screening yet? Why not?” And I get involved too and if it’s getting to the last few weeks* [before QOF census date] *we all tend to chip in.’*

(GP, Practice 8)

However, one optometrist said *they* would be best placed to remind patients but could not do this as screening patients were not registered with them:

*‘If it’s a patient who is* ***our*** *patient, i.e. they normally attend for sight test, we make sure they come; we will phone them ... It is the ones who are for screening only, because we’re not allowed to send them reminders ... we all sit there praying that they will have a screening somewhere, every year.’*

(Screener/optometrist, Programme area 3)

#### 3. Integrating screening with routine care

Routine diabetes care provided opportunities to encourage patients to attend. Staff reminded patients that their screening appointment would be due soon and emphasised the importance of being screened. In Practice 9, receptionists reminded patients who picked up their repeat prescriptions. During screening appointments, nurses who were preparing patients would also pick up on other problems such as elevated blood pressure, thus improving continuity of care. The highest performing practice combined screening with the flu jab:

‘They have to have eye drops put into their eyes and so they usually have a nurse available so that they can have the flu injection and their eye drops put in at the same time, it’s like a conveyor belt really. Then they go and see the man who does the eye inspection.’

(Practice manager, Practice 1)

Screeners again emphasised that practice staff who were aware of the importance of screening and communicated this to their patients could make a difference. However, integrating screening and routine care became problematic in one practice as the nurse felt that involvement in screening took too much of their resources:

*‘*[Screening] *takes up virtually an entire week of my clinical time, which is difficult because I do have other things to be doing than reading people’s eye charts ... the people coming to do the screening should bring somebody with them to do* [acuity tests] *and just do it completely as a unit and not be part of the day-to-day surgeries.’*

(Practice nurse, Practice 6)

#### 4. Focusing on the newly diagnosed

Most practices saw integrating people newly diagnosed with diabetes into the screening programme as important. Practice administrators aimed to add their patients to the DESP lists as soon as possible by letter or fax. Systematic checks of the lists before the annual screening appointment or at the annual QOF audit time were used as a backstop. However, patients could fall through the net:

‘I know several people who say, oh they’ve never been screened before and you say, “can you tell me how long you’ve known about your diabetes?”. And they say “oh a couple of years”. And your heart sinks because sometimes someone has had diabetes for a while before they’re even diagnosed ... so I think that in a way, if we had a better relationship or close relationship with a practice, they perhaps could pick that up.’

(Screener, Programme area 2)

GPs and practice nurses also talked about the need to educate newly diagnosed patients to emphasise the importance of screening and overcome possible anxieties.

#### 5. Staff perceptions of non-attenders

In most practices staff would describe a hard core of ‘difficult to engage’ patients, who would only attend when they began to have symptoms. Patients who did not attend screening were also said to not attend other routine appointments, or to be generally uninterested in their diabetes:

‘Normally there’s this hard core of patients who unless there’s something they need to see a doctor about ... I’ve sat down with the practice nurse and she said “well they won’t turn up, they won’t turn up, they won’t turn up, because they don’t attend for any of their reviews, at all”.’

(Practice manager, Practice 4)

While there was a real sense of frustration with these patients, a perception that some patients are unreachable might lead to staff giving up on trying to motivate them to attend.^16^ However, in three practices (two of which had a high uptake) staff would focus on the practical reasons where people did not attend:

*‘I think a lot of* [non-attenders] *are maybe housebound or workers who just don’t see that diabetes is that much of a problem ... The young chap who was working ... we’ve referred him back to the hospital because his insulin needs sorting out. But the [housebound] older lady who I’m thinking of, I’ve sent I don’t know how many letters and she just …’*

(Practice manager, Practice 3)

### Non-modifiable factors

Population-level factors in the practice’s catchment area, especially deprivation, are recognised as influencing screening uptake. How screening is organised locally, for example the use of high-street optometrists or catch-up clinics, may also impact on uptake.

#### 6. Deprivation

Practices were purposively selected to include deprived areas as this had been shown to make a difference. Two practices situated in locations that were among the 5% most deprived in England had the lowest uptake, but this may have been compounded by a substantial minority ethnic population in the area. For other practices, the link to deprivation was less clear, with the three practices reaching the 80% DESP quality standard located in a mix of more or less deprived areas.

#### 7. Language and ethnicity

Three practices with a high proportion of patients from minority ethnic backgrounds had additional difficulties with screening uptake. One recurring issue was language as written materials sent by the screening programme were in English only. Two South Asian patients said that while they could read the invitation and leaflet, they could imagine difficulties for patients from their mothers’ generation. Practices could partly overcome the language barrier as members of staff spoke the same language as their patients, but often there was a multiplicity of languages spoken in practice areas:

‘We have a mixture of sort of Bengali patients, Punjabi speaking patients. We have a few Somali and Vietnamese type patients ... we’ve got big families with lots and lots of younger children, a lot of families living together, and then we do have obviously the elderly population as well.’

(GP, Practice 9)

Extended families living together as described above could also present practical difficulties: the practice manager in Practice 8 worried about letters going to the wrong patient. In another practice in a largely Sikh area, appointments made in alphabetical order proved impractical:

‘Most of the females are Kaur and most of the males are Singhs. So you will guarantee that the Mrs will turn up and the husband will be with her, and it’s silly for us to then have to send them away because he’s way down on day 3 and she’s on day 2.’

(Practice manager, Practice 6)

The annual screening cycle could lead to some minority ethnic patients falling through the net as they went away to their country of origin for months at a time:

‘People go away ... to the Caribbean, Africa, Asia, Pakistan, India ... You find out in retrospect where they’ve been, and because they’re away they’re not going to get their screening done.’

(GP, Practice 8)

#### 8. Transport and access

Transport was the largest single problematic issue for the two rural practices where public transport was inadequate:

‘In places like this, you know, I mean we don’t have buses that go round the villages and bring people in ... If someone has to have a taxi from [nearby village] to here it’s £30 ... the taxi would wait and then they’d take them home again.’

(Practice manager, Practice 4)

Patients who lived within easy walking/bus distance from the practice and those driven by a spouse or relative were generally satisfied, whereas others found getting home difficult as their vision was still very blurred by the mydriasis drops.

Practice staff and screeners said that patients frequently ignored warnings not to drive, and a few patients admitted having driven home from screening:

‘And then afterwards your eyes are ... you’ve got big pupils and light, oh it’s awful, that’s the thing, you know, you can’t drive home obviously. Um, I know I had it done just before Christmas once and the Christmas decorations were [laughs] as you’re driving down the street you’ve got all these big lights coming at you [laughs] so you have to sort of look down and not look at them.’

(Patient, Practice 2)

In the programme area where patients could make their own appointments with high-street optometrists, there were problems with access as participating optometrists in areas with a high prevalence of diabetes tended to have long waiting lists:

*‘* [Optometrist] *said, it was 3 months, there’s no appointments at all ... because that’s also the nearest one, I said “it’s okay, I’ll wait for three months”. The following year again the same thing ... But the third time ... I chose the one in* [different area] *and straight away she said “I can do it in a couple of hours”. “Isn’t there a waiting list?” She said “no”.’*

(Patient, practice 9)

### Impact of factors in combination on individual practices

No single factor was uniquely represented in practices with high or low uptake rates; however, practices began to struggle when several major barriers came together. For deprived areas, diversity of ethnic backgrounds and languages added to the challenge; for more affluent rural areas, transport was the main barrier. All practices adopted strategies to improve attendance; this was often led by one proactive member of staff who then motivated others to work as a team. In the best performing practices, this strategy worked well. The three practices in the middle struggled as communication with screening teams was fragmented and transport was difficult for patients. In the lowest three practices staff battled against the odds, unable to achieve higher uptake rates.

## DISCUSSION

### Summary

A set of factors that acted as enablers or barriers was identified; while some were related to service and staff interactions, others were related to practice location and demographics. All practices adopted strategies to remind and motivate patients, but the presence of two or more major barriers made it very hard for practices to achieve higher uptake levels. A collaborative and integrated approach with a sense of shared responsibility emerged as integral to improving screening uptake.

### Strengths and limitations

This research took a novel approach to identify the causes of high or low screening uptake with an in-depth case analysis of GP practices. This enabled an analysis of the impact of communication across the interface between patients, practice staff, and regional screening teams. Purposive recruitment meant that practices were diverse, with those in both rural and deprived inner-city areas facing serious challenges. The number of cases were necessarily small; it was not possible to examine all possible permutations between type of location, deprivation, and regionally adopted set-up. As an exploratory case study was conducted, it was not intended to aim for full theoretical saturation; however, every factor identified could be applied across most practices. The only exception was language/ethnicity as only three practices were situated in areas with a substantial minority ethnic population. Additionally, when identifying individual factors possibly linked to uptake, the research team knew uptake rates for practices; this may have influenced their understanding of these factors.

### Comparison with existing literature

The findings confirm results of earlier studies: patients in areas with high deprivation and a large minority ethnic population fared worst. The crucial role played by the accumulation of barriers might explain why, in two recent studies, retinopathy screening uptake was more strongly related to deprivation in Bradford^11^ than in South London.^17^ The importance of health professionals encouraging patients to attend^8^,^9^ was also upheld.

There is little research on the influence of individual practices on screening uptake, although simplifying processes within and between organisations was seen as beneficial in cancer screening.^18^ In this study, communication between services emerged as a major issue, and successful teamwork was seen as vital for increasing uptake. In terms of Boon’s framework for interprofessional teamwork in health care,^19^ practices and screening teams should aim to move from the current model of coordinated teamwork (formalised interaction, for example, through record sharing) to a more integrative model guided by consensus building and a shared vision of patient care.

### Implications for research and practice

In all practices where uptake was less than 80%, a combination of modifiable and non-modifiable factors acted as barriers. It is not possible to pinpoint exactly to what extent they influenced uptake; however, practices already took steps to ameliorate non-modifiable challenges, for example, by using their staff’s language skills to approach minority ethnic patients.^20^

Additional research is needed into the impact of addressing both modifiable and non-modifiable factors, however, this study has identified a range of opportunities to motivate and educate patients that GP practices and screening programmes could explore to increase screening uptake. Strategies should be developed to improve communication between practices and screening teams; and appointments should be flexible enough to enable practices to make a difference. In areas using high-street optometrists, an effort should be made to integrate these into communication networks and ensure availability in areas with a high prevalence of diabetes.

Although the whole practice should link retinopathy with routine diabetes care, study participants also stressed the importance of an identified team leader. This is especially crucial for people newly diagnosed with diabetes. Practices located in challenging areas struggled, however, they also developed strategies to improve uptake, for example, by utilising bilingual members of staff to remind patients face to face or on the phone. Practices should be aware of the existence of information in different languages^21^ and signpost patients to the appropriate leaflets.

The DESP successfully reduced non-attendance rates by implementing centralised call/recall and appointments. In recognition of the disconnect that existed at the time of the study between the organisation of diabetic retinopathy screening and the remuneration for uptake, retinopathy screening will be removed from QOF in 2014–2015. This study’s findings indicated opportunities for improving uptake where screening services and GP practices had effective communication. It is unclear how the new management and remuneration arrangements will affect these important relationships. Practices can use personalised contact with patients likely to miss their appointments and use available technologies, such as phone or text reminders, which have already proven successful for ophthalmology outpatients.^22^ Joint ownership of this challenge is potentially sight saving for many in this hard-to-reach group.

Retinopathy screening by its nature involves several interfaces where communication can be problematic (practice staff, patients, screeners, optometrists, regional screening teams, and increasingly, private providers) and more research is needed into the complexity of these interfaces to make sure that patients do not fall through the net. There is a need for educational interventions for practice staff, which should be robustly evaluated.
